# Ubiquitous urease affects soybean susceptibility to fungi

**DOI:** 10.1007/s11103-012-9894-1

**Published:** 2012-03-01

**Authors:** Beatriz Wiebke-Strohm, Giancarlo Pasquali, Márcia Margis-Pinheiro, Marta Bencke, Lauro Bücker-Neto, Arlete B. Becker-Ritt, Anne H. S. Martinelli, Ciliana Rechenmacher, Joseph C. Polacco, Renata Stolf, Francismar C. Marcelino, Ricardo V. Abdelnoor, Milena S. Homrich, Emerson M. Del Ponte, Celia R. Carlini, Mayra C. C. G. De Carvalho, Maria Helena Bodanese-Zanettini

**Affiliations:** 1 Programa de Pós-Graduação em Genética e Biologia Molecular, Universidade Federal do Rio Grande do Sul (UFRGS), Porto Alegre, Brazil; 2 Programa de Pós-Graduação em Biologia Celular e Molecular, Centro de Biotecnologia, UFRGS, Porto Alegre, Brazil; 3 Biochemistry Department, University of Missouri, Columbia, MO USA; 4 Departamento de Fitossanidade, Faculdade de Agronomia, UFRGS, Porto Alegre, Brazil; 5 Empresa Brasileira de Pesquisa Agropecuária, Embrapa Soja, Londrina, Brazil

**Keywords:** *Glycine max*, Functional analysis, Fungal resistance, Genetic transformation, Overexpression, Co-suppression

## Abstract

The soybean ubiquitous urease (encoded by *GmEu*4) is responsible for recycling metabolically derived urea. Additional biological roles have been demonstrated for plant ureases, notably in toxicity to other organisms. However, urease enzymatic activity is not related to its toxicity. The role of *GmEu*4 in soybean susceptibility to fungi was investigated in this study. A differential expression pattern of *GmEu*4 was observed in susceptible and resistant genotypes of soybeans over the course of a *Phakopsora pachyrhizi* infection, especially 24 h after infection. Twenty-nine adult, transgenic soybean plants, representing six independently transformed lines, were obtained. Although the initial aim of this study was to overexpress *GmEu*4, the transgenic plants exhibited *GmEu*4 co-suppression and decreased ureolytic activity. The growth of *Rhizoctonia solani*, *Phomopsis* sp., and *Penicillium herguei* in media containing a crude protein extract from either transgenic or non-transgenic leaves was evaluated. The fungal growth was higher in the protein extracts from transgenic urease-deprived plants than in extracts from non-transgenic controls. When infected by *P. pachyrhizi* uredospores, detached leaves of urease-deprived plants developed a significantly higher number of lesions, pustules and erupted pustules than leaves of non-transgenic plants containing normal levels of the enzyme. The results of the present work show that the soybean plants were more susceptible to fungi in the absence of urease. It was not possible to overexpress active *GmEu*4. For future work, overexpression of urease fungitoxic peptides could be attempted as an alternative approach.

**Electronic supplementary material:**

The online version of this article (doi:10.1007/s11103-012-9894-1) contains supplementary material, which is available to authorized users.

## Introduction

The soybean plant (*Glycine max*) is affected by several diseases that negatively affect plant yield, eventually resulting in significant crop losses (Sinclair and Hartman 1999). Host genetic resistance is the most desirable and efficient control measure when resistant genotypes are available. However, for some fungal diseases, such as Asian soybean rust, which is caused by *Phakopsora pachyrhizi*, fungicides are the only efficient measure to avoid crop losses. This measure often results in increasing economic and environmental costs (Miles et al. 2007). Understanding the molecular basis of the soybean plant defence against fungal infection and growth, identifying genes involved in hypersensitive or immune response, and characterising their individual roles are key steps for engineering durable and quantitative disease resistance.

Ureases (EC 3.5.1.5) are nickel-dependent metalloenzymes that catalyse the conversion of urea to ammonia and carbon dioxide, thus allowing organisms to use exogenous and internally generated urea as a nitrogen source (Dixon et al. 1975; Krajewska 2009). These enzymes are synthesised by numerous organisms, including plants, fungi and bacteria (Follmer 2008; Krajewska 2009). Two isozymes, which share 87% amino acid identity, have been described for the soybean plant (Goldraij et al. 2003). The embryo-specific urease, encoded by the *GmEu*1 gene (GenBank accession AY230157, Phytozome accession Glyma05g27840.1), is synthesised in the developing embryo and accumulates in mature seeds (Polacco and Havir 1979; Polacco and Winkler 1984; Polacco and Holland 1993), while the ubiquitous urease, encoded by the *GmEu*4 gene (GenBank accession AY230156, Phytozome accession Glyma11g37250.1), is found in lower amounts in all plant tissues (Torisky et al. 1994). The ubiquitous urease is involved in recycling metabolically derived urea (Polacco et al. 1985; Stebbins and Polacco 1995; Witte et al. 2002), but an assimilatory role for the abundant seed urease has not been demonstrated thus far (Carlini and Polacco 2008).

In addition to providing organisms with nitrogen in the form of ammonia, other biological roles have been investigated for plant ureases, especially in toxicity to other organisms. Recent reports confirmed that jackbean (*Canavalia ensiformis*) ureases and the soybean embryo-specific urease display entomotoxic effects (Carlini and Polacco 2008; Carlini et al. 1997) and that purified ureases from jackbean, soybean and cotton seeds inhibit in vitro fungal growth (Becker-Ritt et al. 2007; Menegassi et al. 2008). The toxic activity of the ureases against insects and fungi persisted after urease treatment with irreversible inhibitors of ureolytic activity, demonstrating that other protein domain(s), not the ureolytic active site, are involved in host defence mechanisms (Becker-Ritt et al. 2007; Follmer et al. 2004a, b). The entomotoxic sub-peptide has been identified and cloned from jackbean ureases (Mulinari et al. 2007), while the location of the antifungal domain of ureases has not yet been identified.

Previous studies on the toxic properties of the soybean urease were based on the purified embryo-specific isoform. In the present work, the role of the ubiquitous urease in the soybean response to fungi was investigated in vivo by transcription analyses and the manipulation of *GmEu*4 gene expression in transgenic soybean plants.

## Experimental procedures

### *P. pachyrhizi* bioassay for gene expression analysis

The reaction of soybean plants to rust infection was assessed by the inoculation of *P. pachyrhizi* spores collected in the field into plants maintained under greenhouse conditions at Embrapa Soja, Londrina, PR, Brazil. The soybean plants were grown in a pot-based system and maintained in a greenhouse at 28 ± 1°C with 16/8 h light/dark at a light intensity of 22.5 μEm^−2^ s^−1^. The Embrapa-48 genotype, which develops a Tan lesion (van de Mortel et al. 2007), was used as the susceptible standard and the PI561356 genotype, which carries the resistance to soybean rust mapped to linkage group G, was used as the resistant standard (Camargo 2010). Uredospores were harvested from leaves exhibiting sporulating uredia and diluted in distilled water with 0.05% Tween-20 to a final concentration of 3 × 10^5^ spores/mL. The spore suspension was sprayed onto plantlets at the V2 developmental stage. The same solution lacking spores was used for mock inoculations. Following fungal or mock inoculations, water-misted bags were placed over all plants for 1 day to promote infection and to prevent cross-contamination of the mock-infected plants. One trifoliate leaf from each plant was collected at 1, 12, 24, 48, 96 and 192 h after inoculation, frozen in liquid nitrogen, and stored at −80°C. Three biological replicates from each genotype were analysed for both treatments.

### Plasmid construction

**Fig. 1:**
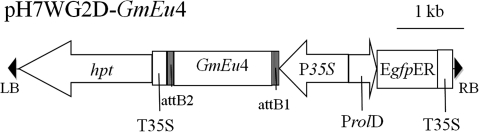
T-DNA region of binary vector pH7WG2D-*GmEu*4 used for soybean transformation. *RB* T-DNA right border, *LB* left border, *hpt* hygromycin phosphotransferase gene, *P35S* Cauliflower mosaic virus (CaMV) 35S promoter, *T35S* CaMV 35S terminator, *EgfpER* enhanced green fluorescent protein, *P*rolD root loci D promoter, *GmEu*4 soybean ubiquitous urease enconding gene, *attB1 and attB2* LR reaction site, *kb* kilobase pairs (1,000 bp)

The plasmid pGPTV-JIT, containing the ubiquitous urease cDNA was kindly provided by Dr. Mark Taylor (Scottish Crop Research Institute, Dundee, Scotland). This vector was used as template for PCR amplification. The PCR mixture consisted of 100 ng of template DNA, 0.2 mM of dNTPs, 0.5 μM of each primer (5′-CACCTTAAAAATGAAACTG-3′ and 5′-TAAAAGAGGAAGTAATTTCG-3′), 1× *Pfu* Buffer, 2.5 U of *Pfu* DNA Polymerase (Fermentas, Glen Burnie, USA) and autoclaved distilled water in a total volume of 50 μL. The reactions were heated in the beginning (5 min at 94°C) and subjected to 35 cycles as follows: 1 min at 94°C, 1 min at 42°C, and 3 min at 72°C. The Gateway^®^ System (Invitrogen, Carlsbad, USA) was used to clone the PCR product into the pH7WG2D vector (Karimi et al. 2002) for *GmEu*4 overexpression. The T-DNA region of the resulting pH7WG2D-*GmEu*4 vector contained the *GmEu*4 gene ORF under control of the CaMV *35S* promoter, the hygromycin-phosphotransferase marker gene (*hpt*), and the green fluorescent protein reporter gene (*gfp*) (Fig. 1). The pH7WG2D-*GmEu*4 vector was transformed into *Agrobacterium tumefaciens* LBA4404 for plant transformation.

### Plant transformation and regeneration

Seeds from soybean cultivars IAS5 and Bragg were supplied by Embrapa Soja, Londrina, PR, Brazil. Pods containing immature seeds of 3–5 mm in length were harvested from field grown plants. Somatic embryogenesis was induced from immature cotyledons and proliferated as described by Droste et al. (2002).

Eight-month-old proliferating embryogenic tissues were submitted to transformation by particle bombardment using the particle inflow gun (PIG) (Finer et al. 1992) according to the procedure described by Droste et al. (2002) or by the combined DNA-free particle bombardment and *Agrobacterium* system as previously described (Wiebke-Strohm et al. 2011). Seven dishes with 15 embryogenic clusters/dish, with approximately 0.67 mg/cluster, were prepared for bombardment, and 10 dishes were used in the bombardment/*Agrobacterium* transformation experiment. After three months in hygromycin-B selection medium, hygromycin-resistant embryogenic soybean tissues were visually selected, counted and individually cultured for the establishment of lines corresponding to independent transformation events.

Embryo histodifferentiation, conversion into plants and acclimation were carried out as described by Droste et al. (2002). All plants derived from an independent piece of hygromycin-resistant tissue were noted as being cloned plants. Plants derived from non-transformed embryogenic tissues submitted to the same culture conditions were recovered and used as controls for molecular characterisation and bioassays.

For progeny analysis, seeds obtained from T_0_ plants were planted in pots containing 1 kg of organic soil and grown in the greenhouse under the same conditions presented above. The plants were supplemented with fertilizers every 15 days.

### PCR and GFP expression screening for transgenic plants

Total DNA was extracted from plant leaves as previously described (Doyle and Doyle 1987). Putative transgenic plants were PCR-screened for the presence of the hygromycin resistance gene (*hpt*) and a chimeric gene (P*35S*-*GmEu*4) formed by the CaMV *35S* promoter (P*35S*) and the ubiquitous urease ORF (*GmEu*4). The following primer pairs were used in the PCR assays: 5′-GAGCCTGACCTATTGCATCTCC-3′ and 5′-GGCCTCCAGAAGAAGATGTTGG-3′ (*hpt*); 5′-CGCACAATCCCACTATCCTT-3′ and 5′-ATGCTAGTTCAAGGTTTCCATTCT-3′ (P*35S*-*GmEu*4). The PCR mixture consisted of 200 ng of template DNA, 0.4 mM of dNTPs, 0.4 μM of each *primer*, 2.5 mM of MgCl_2_, 1× Taq Buffer, 1 U of Taq DNA Polymerase (Invitrogen, São Paulo, Brazil), and autoclaved distilled water in a final volume of 25 μL. The reactions were heated at the beginning (5 min at 94°C) and subjected to 30 cycles as follows: 45 s at 94°C, 45 s at 42°C and 45 s at 72°C. After electrophoresis in a 1% agarose gel containing ethidium bromide (0.01 mg/L), the PCR products were visualised under ultraviolet light.

GFP expression was detected under blue light using an Olympus^®^ fluorescence stereomicroscope equipped with a BP filter set containing a 488 nm excitation filter and a 505–530 nm emission filter. Images were captured using the software QCapture Pro™ 6 (QImaging^®^).

### Reverse transcription, quantitative (real-time) PCR (RT-qPCR)

Total RNA was extracted using the TRIzol reagent (Invitrogen, Carlsbad, USA) and further treated with DNAse I (Promega, Madison, USA) according to the manufacturer’s instructions. First-strand cDNAs were obtained with approximately 2 μg of DNA-free RNA in the M-MLV Reverse Transcriptase System™ (Invitrogen, Carlsbad, USA) with a 24-polyVT primer.

RT-qPCR was conducted in a StepOne Applied Biosystem Real-time Cycler™. The PCR-cycling conditions were implemented as follows: 5 min at 94°C, followed by 40 repetitions of 10 s at 94°C, 15 s at 60°C and 15 s at 72°C, and ending with 2 min at 40°C. A melting curve analysis was performed at the end of the PCR run over a range of 55–99°C, increasing the temperature stepwise by 0.1°C every 1 s. Each 25 μL reaction comprised 12.5 μL of diluted DNA template, 1 × PCR buffer (Invitrogen, São Paulo, Brazil), 2.4 mM of MgCl_2_, 0.024 mM of dNTPs, 0.1 μM of each primer, 2.5 μL of SYBR-Green (1:100,000, Molecular Probes Inc., Eugene, USA) and 0.3 U of Platinum Taq DNA Polymerase (Invitrogen, São Paulo, Brazil). Two different templates were evaluated: a first-strand cDNA-reaction product (1:100) for relative expression analyses and genomic DNA (1:100, 1:1,000 e 1:10,000) for gene copy number estimation. All PCRs were carried out in technical quadruplicates. Reactions lacking template were used as negative controls.

PCR amplifications were performed using gene-specific primers (Table 1). Primer pairs designed to amplify an F-Box protein, a Metalloprotease and the Actin 11 sequences were used as internal controls to normalise the amount of mRNA present in each sample, whereas a primer pair for a Lectin gene was used as a reference for DNA amplification in gene copy number estimations. These genes were confirmed as good reference genes in previous reports (Jian et al. 2008; Libault et al. 2008; Schmidt and Parrott 2001). All expression data analyses were performed after comparative quantification of amplified products using the 2^−ΔΔCt^ method as previously described (Livak and Schmittgen 2001). The transgene copy number was estimated by relative quantification after a standard curve analysis as previously described (Shou et al. 2004).

**Table 1:** Primer set designed for RT-qPCR

Target	Orientation	Primer sequence	Efficiency of primer (%)	PCR product size (bp)
Endogenous plus transgenic ubiquitous urease transcripts or DNA quantification	Forward	5′-TGGTGATCAAAGGTGGTGAG-3′	104.6	121
	Reverse	5′-GAACTACCAGCCTTGCCAAA-3′		
Endogenous ubiquitous urease transcripts	Forward	5′-TCACTGTGGACCCAGAAACA-3′	99.65	160
	Reverse	5′-CTTGCTTATTGTTTTTTGCCAAT-3′		
Actin 11 transcripts	Forward	5′-CGGTGGTTCTATCTTGGCATC-3′	98.04	142
	Reverse	5′-GTCTTTCGCTTCAATAACCCTA-3′		
Metalloprotease transcripts	Forward	5′-ATGAATGACGGTTCCCATGTA-3′	99.35	114
	Reverse	5′-GGCATTAAGGCAGCTCACTCT-3′		
F-Box transcripts	Forward	5′-AGATAGGGAAATGTTGCAGGT-3′	99.44	93
	Reverse	5′-CTAATGGCAATTGCAGCTCTC-3′		
PR4 transcripts	Forward	5′-AACCTTACTCATGGCGCAGT-3′	ND*	150
	Reverse	5′-TGCTGCACTGATCTACGATTC-3′		
Lectin DNA quantification	Forward	5′-TACCTATGATGCCTCCACCA-3′	106.2	129
	Reverse	5′-GAGAACCCTATCCTCACCCA-3′		

* *ND* non-determined

### Ureolytic activity

The ureolytic activity in transgenic and control plants was evaluated by determining the ammonia released by enzymatic activity. First, for visual estimation, five leaf discs (0.5 cm in diameter) per plant were incubated in 1 mL of a urease indicator solution for 24 h at 60°C as previously described (Meyer-Bothling and Polacco 1987). 1 L of urease indicator solution was prepared with 6 g of urea, 10 mL of cresol red (1 mg/mL), 10 mL of KH_2_PO_4_/K_2_HPO_4_/EDTA, pH 7.0 and 1 mL of azide 20% (w/v). Subsequently, for enzymatic activity quantification, the protein was extracted from powdered leaves and the protein content in the crude extract was determined by the method reported by Bradford (1976) using bovine serum albumin as a standard. The protein crude extracts were incubated with 10 mM of urea in 10 mM of sodium phosphate, pH 7.5, for 45 min at 37°C, and the ammonia released was measured colorimetrically as previously described (Weatherburn 1967). Each sample was tested four times. One unit of urease releases 1 μmol of ammonia per min, at 37°C, pH 7.5.

### Fungal bioassays

Powdered leaves (1 g) were resuspended in 5 mL of 20 mM of a sodium phosphate buffer (NaPB) containing 1 mM of EDTA and 2 mM of β-mercaptoethanol, pH 7.5. The protein content in the crude extracts was determined by the method reported by Bradford (1976) using bovine serum albumin as a standard.

The growth of soybean fungal pathogens *R. solani* and *Phomopsis* sp., as well as the soybean non-pathogen, *P. herguei*, was evaluated turbidimetrically according to a previously described method (Becker-Ritt et al. 2007). Briefly, 10 μL of a spore suspension (100 spores/μL) were inoculated onto 96-well plates containing 110 μL of Potato Dextrose Broth (PDB, Becton Dickenson Co.), pH 7.0 and incubated at 28°C. After 16 h, 15 μg of crude protein extract, diluted in 50 μL of NaPB buffer, was added to the fungal suspension (0 h). The plates were incubated at 28°C, and the absorbance (430 nm) was determined on a plate reader (Spectramax, Molecular Devices) at 0, 24, 36, 48 and 60 h after protein addition. Fungal growth at 24, 36, 48 and 60 h was calculated relative to the 0 h absorbance, which was considered to be one. NaPB buffer and 9.5% hydrogen peroxide (H_2_O_2_) were used as controls. Three samples/plant/fungus were prepared.

A detached leaf method was used to evaluate soybean plant infection by *P. pachyrhizi* (Twizeyimana et al. 2006). Fully expanded trifoliate leaves from 2-month-old plants were collected, rinsed in sterile distilled water and cut in 5 cm × 5 cm pieces. Each leaf piece was inoculated by dripping 1 mL of a uredospore suspension (10^5^ spores/mL) and placed with the abaxial side upwards in a Petri dish covered with wet filter paper. The plates were incubated at 20°C with a 12/12 h light/dark cycle. The disease symptom development was recorded 12 days after inoculation.

### Statistical analysis

The relative expression levels of *GmEu*4 in soybeans during *P. pachyrhizi* infection were statistically compared by variance analysis with three-factor factorial treatments: genotypes, time and pathogen presence. The data were transformed using the weighted least squares method. The means were compared using a Bonferroni multiple comparison test. The Chi-squared (χ^2^) test was used to confirm the Mendelian expected transgene segregation pattern in the progeny (3:1 transgenic: non-transgenic plants). The data from filamentous fungal bioassays were analysed using one-way ANOVA. When necessary, data were transformed using the weighted least squares method. A Bonferroni multiple comparison test was performed to compare the treatments. A non-parametric *t* test was carried out to compare the effect of *P. pachyrhizi* on transgenic and non-transgenic plants. The results with *p* ≤ 0.05 were considered significant.

## Results

### *GmEu*4 expression in response to *P. pachyrhizi* infection

**Fig. 2:**
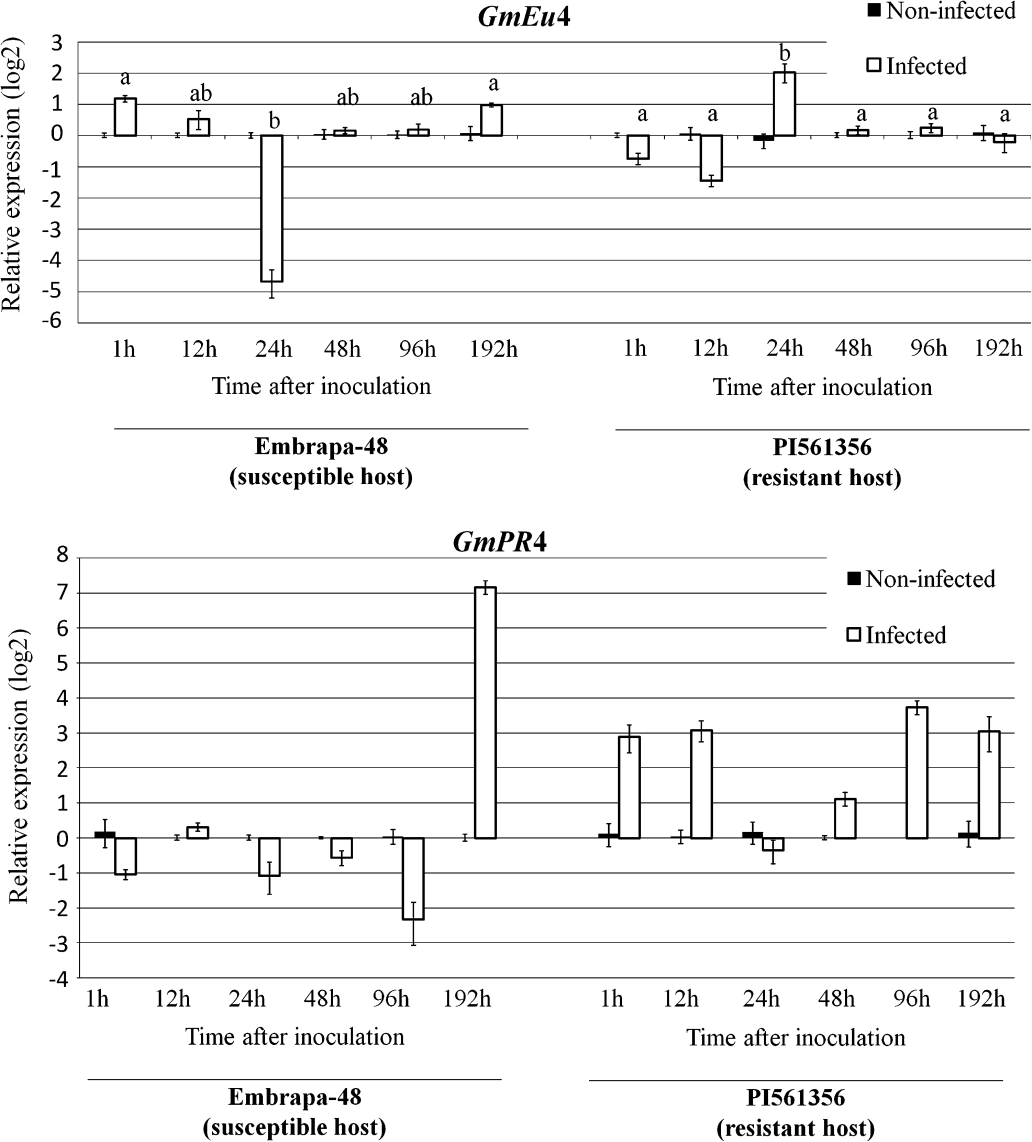
*GmEu*4 and *GmPR*4 gene expression in response to *P. pachyrhizi* infection in susceptible and resistant soybean genotypes. The relative expression levels of *GmEu*4 and *GmPR*4 in the soybean leaves of susceptible (Embrapa-48) and resistant (PI561356) genotypes were determined by RT-qPCR 1, 12, 24, 48, 92 and 196 h after *P. pachyrhizi* (infected) or mock (non-infected) inoculation. In these *panels*, the *y*-axes indicate that the scale is log2 (fold-change) to allow a better comparison of groups of genes presenting large differences in their expression levels. The values are the means of three biological replicates with four technical replicates each. The means followed by equal letters in the same cultivar do not differ significantly (Bonferroni multiple comparison test, *p* < 0.05). The F-Box protein and Metalloprotease reference genes were used as internal controls to normalise the amount of mRNA present in each sample. The transcript levels of *GmEu*4 and *GmPR*4 in mock-inoculated plants were used to normalise the transcript accumulation in inoculated plants

The transcript levels of *GmEu*4 in soybeans inoculated with *P. pachyrhizi* were determined by RT-qPCR. The leaves of two soybean genotypes exhibiting contrasting responses to rust were assayed: Embrapa-48 is highly susceptible, while PI561356 is more resistant (van de Mortel et al. 2007; Camargo 2010). Differences in interactions among genotypes, different points in the time-course and the presence or absence of pathogens were highly significant (*p* < 0.0001). *GmEu*4 expression in non-infected (mock) plants was continuous during the infection for both genotypes. As shown in Fig. 2, in the susceptible host, *GmEu*4 transcripts were significantly upregulated (2.26-fold) at 1 h, followed by a strong downregulation (25-fold) at 24 h and another upregulation peak at 192 h after pathogen inoculation. In contrast, in the resistant genotype, *GmEu*4 expression was strongly upregulated (4.07-fold) 24 h after the inoculation.

The host response to infection was confirmed by *GmPR*4 (GenBank accession Z11977, Phytozome accession Glyma19g43460.1) expression. This gene encodes a wound-induced protein, which has been shown to accumulate in wounded and *Phytophthora sojae* elicitor-treated soybean tissues (Graham et al. 2003). As shown in Fig. 2, PI561356 leaves rapidly and consistently increased *GmPR*4 mRNA levels after infection, while the susceptible Embrapa-48 leaves increased mRNA levels only 192 h after inoculation. A biphasic and earlier *GmPR*4 expression was observed in the resistant genotype. A similar expression profile has been described for many other genes involved in the plant defence system (Panthee et al. 2007; Soria-Guerra et al. 2010a, b; van de Mortel et al. 2007).

### Generation and characterization of transgenic plants

To determine whether *GmEu*4 overexpression would enhance resistance to fungi, soybean somatic embryos were transformed with the pH7WG2D-*GmEu*4 vector. The efficiency of plant recovery via somatic embryogenesis is genotype dependent (Droste et al. 2002); therefore, the cultivars IAS5 and Bragg were chosen for this experiment. A total of twenty-nine well-developed plants, representing six independent transgenic lines, were regenerated from two transformation experiments. Twenty-six plants from cultivar IAS5, derived from four independent transgenic lines, were recovered from tissue submitted to particle bombardment. Three plants were recovered from embryos transformed by bombardment/*Agrobacterium*. These plants correspond to two independent transgenic lines, one from cultivar IAS5 and one from Bragg (Table 2). All of the plants flowered, and 21 set seeds.

**Table 2:** Number of transgenic soybean adult plants and independent lines

	Experiment I (bombardment)		Experiment II (bombardment/*Agrobacterium*)	
	IAS5	Bragg	IAS5	Bragg
Adult plants				
Independent lines	4	–	1	1
Plants	26	–	1	2
Plants with seeds				
Independent lines	3	–	–	–
Plants	21	–	–	–

The cultivars IAS5 and Bragg were selected to transformation experiment due to their competence to somatic embryogenesis. Transformation experiments were carried out by bombardment and bombardment/*Agrobacterium*

Stable transgene integration was confirmed in all plants by PCR using primers for *hpt* and *GmEu*4 (one specific to the CaMV *35S* promoter and another specific to the *GmEu*4 ORF) (data not shown). Furthermore, transgenic embryogenic tissues and plants were green fluorescent under blue light (Supplementary Fig. 1). The DNA of one plant from each independent transformed line was assayed by qPCR to determine the number of transgenic insertions (Shou et al. 2004; Yuan et al. 2007). PCR efficiencies were similar for *GmEu*4 (104.6%) and lectin (106.2%), allowing the use of lectin as a reference gene for qPCR. The number of recombined *GmEu*4 copies in transgenic plant genomes was calculated proportionally to those of non-transgenic plants. A non-transgenic soybean genome contains one *GmEu*4 gene (two alleles in diploid genomes). The number of recombined *GmEu*4 copies in the genome of transgenic soybean plants varied from 1 to 14 (Table 3). As expected, bombardment-derived plants exhibited a higher number of extra *GmEu*4 copies (more than 10), while lower numbers were found in *Agrobacterium*-derived lines (one).

**Table 3:** Number of recombinant *GmEu*4 copies integrated into transgenic plant genome. Estimative was performed by qPCR comparing DNA quantification of transgenic plants and non-transgenic plants

	Cultivar	Transformation line	Recombined *GmEu*4 copies
Bombardment	IAS5	A1	11 ± 2
		A2	ND*
		A3	13 ± 3
		A8	14 ± 2
Bombardment/*Agrobacterium*	IAS5	4F	1 ± 0.1
	Bragg	7E	1 ± 0.1

Lectin was used as reference gene

* *ND* not determined

**Fig. 3:**
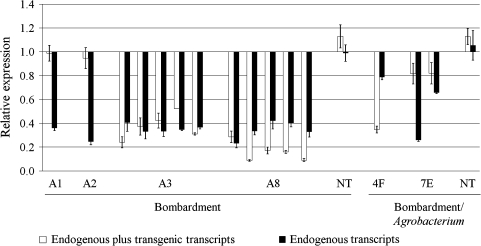
Endogenous and transgene *GmEu*4 expression pattern in transgenic and non-transgenic (control) plants. Six *transgenic lines* (A1, A2, A3, A8, 4F and 7E) were evaluated. Five plants derived from *lines* A3 or A8, two plants from *line* 7E and one plant each from *lines* A1, A2 and 4F were analyzed. NT represents the mean ± SD of four non-transformed plants. Ubiquitous urease transcripts were detected by two different primer pairs: one for endogenous plus transgenic transcripts (hybridising to the encoding region) and another for endogenous transcripts (hybridising to the 3′ UTR region). The F-Box protein, Metalloprotease and Actin 11 reference genes were used as internal controls to normalise the amount of mRNA present in each sample. The transcript level of *GmEu*4 present in non-transformed plants was used to normalise the transcript accumulation in transgenic plants

The expression of endogenous and recombined *GmEu*4 genes was evaluated by RT-qPCR using two different primer pairs: one for endogenous plus transgenic transcripts (encoding region) and another for endogenous transcripts only (3′ UTR region) (Fig. 3). As expected, independent of the primer pair used, the same transcript levels were observed in non-transformed plants, which contain only the endogenous gene. The expression levels were also equivalent in both IAS5 and Bragg soybean cultivars (data not shown). In transgenic plants, a drastic variation in *GmEu*4 mRNA levels was observed when different primer pairs were used in RT-qPCR, which may reflect transgene expression effects. Surprisingly, most transgenic plants (A3, A8, 4F and 7E lines) showed lower *GmEu*4 expression than non-transformed controls. For two other transgenic plants (A1 and A2 lines), the transcript accumulation was equivalent to wild-type plants. The expression pattern of endogenous *GmEu*4 was similar among plants of different transgenic lines. The endogenous transcripts were downregulated in the presence of a recombined *GmEu*4 copy(ies). This phenomenon is termed “co-suppression” (Napoli et al. 1990), and in the present work, co-suppression refers to simultaneous endogenous and transgene silencing.

Although the initial aim of this study was to overexpress *GmEu*4, the co-suppressed plants represent a powerful tool for analysis of gene function because null mutants have never been obtained for the ubiquitous urease (Carlini and Polacco 2008). The *eu*4 mutant produces a protein with a deficient ureolytic activity. However, the *eu*4 mutant was not useful for study of entomo- or fungitoxicity because urease enzymatic activity is not related to these properties (Becker-Ritt et al. 2007; Follmer et al. 2004a, b). Urease-deprived soybean plants were obtained in the present study by urease co-suppression. Thus, further experiments were carried out with A3 and A8 lines, which exhibited a higher degree of *GmEu*4 co-suppression.

**Fig. 4:**
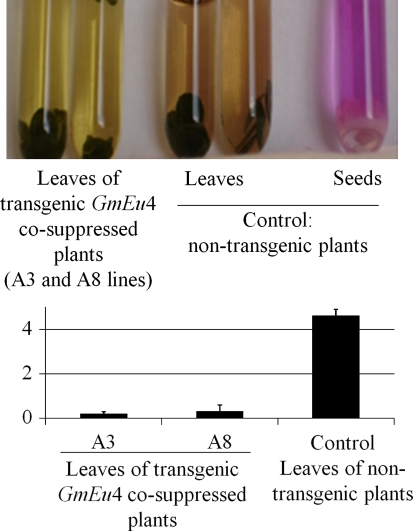
Ureolytic activity in *GmEu*4 co-suppressed plants. In the *upper panel*, leaf discs or seed chips were incubated in a pH-indicator reagent made of cresol *red* in the presence of weakly buffered 10 mM urea (Meyer-Bothling and Polacco 1987). As the ureolytic activity proceeds, the released NH_4_^+^ increases the pH, turning the solution from *yellow* to *pinkish* and finally to a deep vermillion. In the *lower panel*, phenol-hypochlorite was used to determine the amount of liberated ammonium catalysed by the protein crude extracts. One unit of urease releases 1 μmol of ammonia per minute at 37°C, pH 7.5. The bars represent the mean ± SD of three independent experiments carried out with nine plants from the A3 *line*, seven plants from the A8 *line* and 5 non-transformed plants. The *symbol* * indicates that values are significantly different by a Student’s *t* test, *p* < 0.05

Changes in the ureolytic activity confirmed the urease co-suppression in the transgenic soybean plants (Fig. 4). The activity was determined by the semi-quantitative seed chip/leaf peace assay (Meyer-Bothling and Polacco 1987), in which either a leaf piece or a sliver taken non-destructively from a mature seed is placed in a solution of urea that has been weakly buffered at pH 7.0 and contains cresol red as pH indicator. Because urea is hydrolysed by urease in the tissue sample, increasing pH (due to consumption of H^+^ in urea conversion to 2 NH_4_^+^ and HCO_3_^−^) converts the cresol red from yellow, at neutrality, to pink or red. For non-transformed seeds (positive control), the solution became pink in 1 min. Leaves of non-transformed plants produced a light pink colouration after 8 h of incubation, in agreement with the dramatically lower urease levels found in leaves compared to seeds (Torisky et al. 1994). No colour change was observed in solutions containing leaves of co-suppressed plants, even after 24 h of incubation. The reduced urease activity of transgenic plants was confirmed by a quantitative method using leaf protein crude extracts (Fig. 4). When compared to the non-transgenic plants, drastically lowered ureolytic activity was observed in urease co-suppressed A3 and A8 lines.

### Transgenic progeny

Progeny were successfully obtained from three T_0_ transgenic lines (A1, A3 and A8). Twenty plants, derived from each original line, were evaluated for transgene segregation by PCR and GFP expression analyses (Table 4). The data confirmed transgene stability and fit the expected 3:1 Mendelian segregation ratio for a single dominant locus. This result is not unexpected because particle bombardment often generates very large, high copy number transgenic loci (Kohli et al. 2003; Yin et al. 2004). The integration of multiple copies into a single locus was previously observed in other transgenic soybean plants generated by the same method (Homrich et al. 2008; Schmidt et al. 2008). Fourteen T_1_ PCR-positive plants from A3 and A8 families were analysed by RT-qPCR for transgene expression, and most of them maintained the co-suppressed phenotype (data not shown).

**Table 4:** Transgene segregation in three T_1_ families of *GmEu*4 transgenic soybean plants

T_0_ line	Total of T_1_ analyzed plants	T_1_ plants PCR/GFP+	T_1_ plants PCR/GFP−	Expected ratio	p*
IAS5 A1	20	14	6	3:1	0.72
IAS5 A3	20	11	9	3:1	0.18
IAS5 A8	20	12	8	3:1	0.31
IAS5 NT	20	0	20	–	–

*Transgene segregation ratios were tested by χ^2^ non-parametric test

### Bioassays with filamentous fungi

The effects of the ubiquitous urease on filamentous fungal vegetative growth were determined by a turbidimetric assay. Two soybean fungal pathogens (*R. solani* and *Phomopsis* sp.) and one maize pathogen (*P. herguei*) were included in the test because their sensitivity to purified *C. ensiformis* and embryo-specific soybean ureases has been previously reported (Becker-Ritt et al. 2007).

**Fig. 5:**
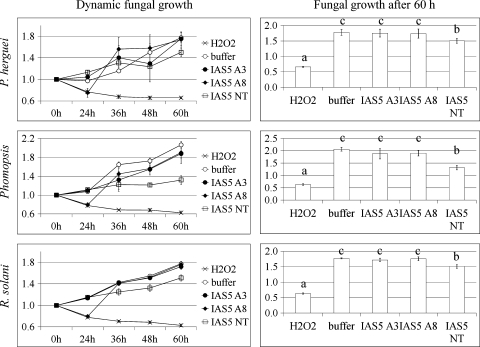
Fungal growth after 60 h on soybean crude extracts of *GmEu*4 co-suppressed plants. Spores (1,000 spores in 10 μL) were inoculated into 110 μL PDB, incubated at 28°C for 16 h and subsequently, crude leaf protein extract (15 μg in 50 μL) was added to the fungal culture. The samples were incubated at 28°C and the absorbance (430 nm) was recorded every 12 h. The experiment was carried out with four T_0_ co-suppressed plants (two from each A3 and A8 *lines*) and three non-transformed plants (IAS5 NT). Hydrogen peroxide (H_2_O_2_) and protein extraction buffer were taken as positive and negative controls of the experimental conditions, respectively. For biological interpretations, transgenic and non-transgenic plants of the same cultivar must be compared. Technical triplicates were analysed per sample. The data (mean ± SD) are proportional to the 0 h absorbance, which was considered to be one. The means followed by different letters in the same fungus/experiment are significantly different (ANOVA, Bonferroni multiple comparison test, *p* < 0.05)

Increasing turbidity of the medium, as the result of hyphal development, was observed over time (Fig. 5). H_2_O_2_ and protein extraction buffer were used as positive and negative controls for the experimental conditions, respectively. However, to evaluate the biological role of urease on fungal growth, the results obtained from transgenic and non-transgenic plants of the same cultivar must be compared because urease content is supposed to be the unique distinctive characteristic between these plants. *P. herguei*, *Phomopsis* sp. and *R. solani* growth in protein extracts from two independent transgenic co-suppressed plants was significantly higher than in that from non-transgenic plants. This result suggests that the urease-deprived plants were more susceptible to fungal growth than the plants with normal levels of urease.

### Bioassays with *P. pachyrhizi*

**Fig. 6:**
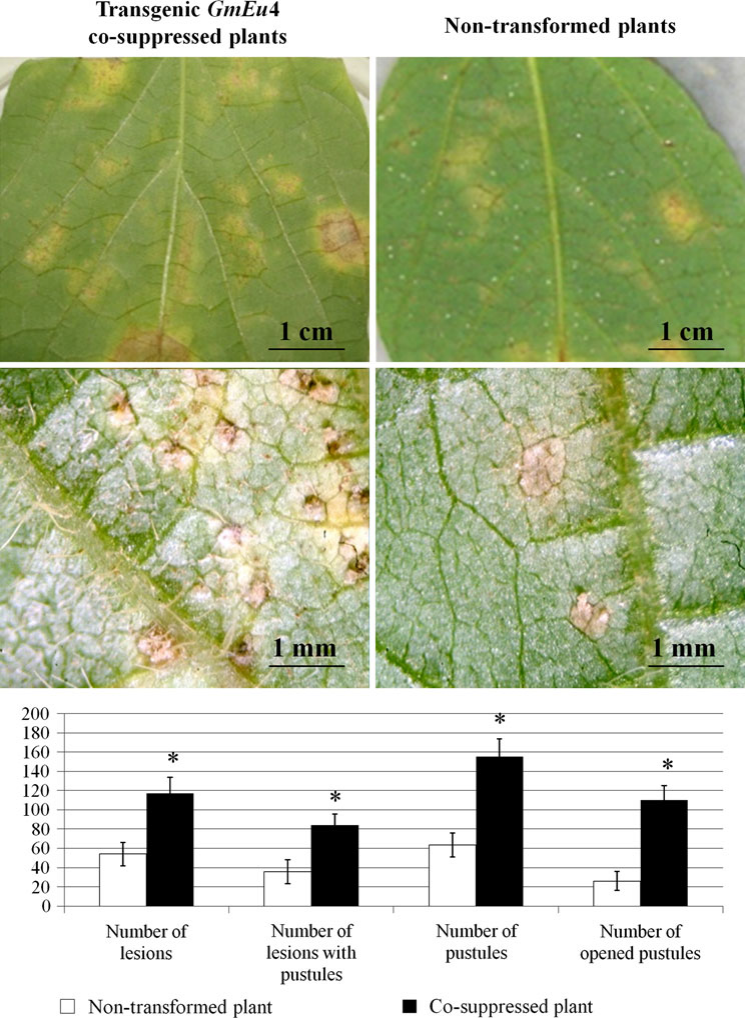
Soybean rust (*P. Pachyrhizi*) development on detached soybean leaves of *GmEu*4 co-suppressed plants 12 days after inoculation. Detached leaves were inoculated with 10^5^/mL of a uredospore suspension and incubated at 20°C. In the *upper panel*, *Tan-coloured* lesions and pustules as observed under stereomicroscope are shown. In the *lower panel*, the evaluation of the four infection parameters were evaluated in the seven T_1_ co-suppressed plants (four from A3 *line* and three from A8 *line*) and three non-transgenic plants is shown. The *symbol* * indicates that the means are significantly different between leaves of transformed and non-transformed plants (Student’s *t* test, *p* < 0.05)

Tan lesions were observed on all detached leaves 12 days after *P. pachyrhizi* inoculation (Fig. 6). However, the number of lesions, number of lesions with pustules, number of pustules and number of opened pustules were significantly higher in *GmEu*4 co-suppressed lines (Fig. 6). The susceptibility of the detached leaves to *P. pachyrhizi* is in agreement with previous results using intact plants (Twizeyimana et al. 2006). It is important to note that no visible difference was observed in the timing of lesion and pustule formation or pustule eruption (data not shown).

## Discussion

### Generation of transgenic soybean plants exhibiting lower levels of ubiquitous urease

The present work aimed to obtain transgenic soybean plants overexpressing the *GmEu*4 gene. However, most of the recovered transgenic plants exhibited simultaneous co-suppression of the endogenous *GmEu*4 gene and the transgene construct. The reduction in ureolytic activity, ascribed to decreased enzyme accumulation, confirmed the co-suppression. Co-suppressed plants were derived from six independent transformed lines, containing one to 14 extra copies of recombinant *GmEu*4.

The co-suppression phenomenon is not uncommon in transgenic plants (Napoli et al. 1990; El-Shemy et al. 2006; Francis and Spiker 2005; Tang et al. 2007). Several mechanisms have been proposed to explain the co-suppression. The effects of transgene copy number on the level of gene expression are known to be complex and could in part explain our results. Though the increase of transgene copy number is expected to enhance the expression level (El-Shemy et al. 2006), there is evidence that gene co-suppression is frequently associated with multiple inserts (James et al. 2002; Lechtenberg et al. 2003; Tang et al. 2007). Alternatively, Francis and Spiker (2005) suggested that in silenced transgenic lines, T-DNAs may have integrated into genomic regions that repress transgene expression.

In addition, urease is involved in ammonia release (Dixon et al. 1975; Krajewska 2009) and is toxic to pathogenic and pest organisms (Carlini and Polacco 2008; Carlini et al. 1997; Becker-Ritt et al. 2007; Menegassi et al. 2008). The high number of transgenic lines with gene co-suppression obtained in this study demonstrates that overexpression of the complete *GmEu*4 ORF is challenging. Torisky et al. (1994) identified the *GmEu*4 gene by complementation of a mutant and were able to obtain overexpression of this gene in only 20% of transgenic calluses. These transgenic calluses were not successfully converted into plants. Together, these results suggest that plants may have a strong regulation system to avoid the accumulation of urease.

In the present study, we were not able to ascribe the co-suppression observed to any of the mechanisms described above. The precise determination of the cause of co-suppression in a particular transgenic line is still largely an empirical problem and requires in-depth analyses of the individual transgenic lines.

### Levels of ubiquitous urease in soybean affect the development of fungi

It has been previously demonstrated that purified ureases from soybean (embryo-specific urease), jackbean and cotton seeds display antifungal properties (Becker-Ritt et al. 2007; Menegassi et al. 2008). The results of the present study consistently demonstrated that the absence of the *GmEu*4 product enhanced susceptibility of the soybean plant to necrotrophic pathogens, *P. herguei*, *Phomopsis* sp. and *R. solani*, and a biotrophic pathogen, *P. pachyrhizi*. As observed for other ureases, the soybean ubiquitous urease affected the development of a broad spectrum of fungi. In addition, it was confirmed that the in vivo urease levels influenced the plant susceptibility to necrotrophic and biotrophic fungal pathogens.

Contrasting *GmEu*4 expression between resistant and susceptible genotypes was most evident 24 h after *P. pachyrhizi* inoculation, when an upregulation peak was observed in the resistant host (PI561356) while strong downregulation occurred in the susceptible host (Embrapa-48). The initial infection process, common to resistant and susceptible hosts, occurs within the first 16–24 h (Goellner et al. 2010). At this point, hyphal growth proceeds and haustoria become visible only in susceptible hosts. In addition, in the present work, the co-suppressed *GmEu*4 plants developed a significantly higher number of lesions, pustules and erupted pustules than the susceptible non-transformed plants, suggesting a more intensive colonisation by the pathogen.

Our results suggest that the ubiquitous urease may directly or indirectly inhibit fungal development and we hypothesise that urease interferes with the hyphae osmotic balance. Urease interference in fungal osmotic balance has been proposed by Becker-Ritt et al. (2007). Scanning electron microscopy demonstrated that *P. herguei* exhibited cell wall damage when grown in vitro in the presence of purified soybean embryo-specific or jackbean urease (Becker-Ritt et al. 2007). Recently, it was shown that in vitro jackbean purified urease is able to permeabilise liposomes (Barros et al. 2009) and planar lipid bilayers (Piovesan A., personal communication). Further experiments comparing the fungal growth in transgenic and non-transgenic plants will help to elucidate the mechanism of the urease fungitoxic effect.

Recently, large-scale transcript profiles of soybean plants during *P. pachyrhizi* infection have been published (Choi et al. 2008; Panthee et al. 2007, 2009; Soria-Guerra et al. 2010a, b; van de Mortel et al. 2007). These studies were carried out using different resistant and susceptible genotypes of *G. max* and *Glycine tomentella*. The differences in gene expression between the susceptible and resistant host peaked at 12 and 72 h post inoculation (Panthee et al. 2007; Soria-Guerra et al. 2010a; van de Mortel et al. 2007). Additionally, it has been demonstrated that the host phenological stage during *P. pachyrhizi* inoculation affects the gene expression pattern (Panthee et al. 2009). In fact, many reports suggest that the timing and the degree of induction of a defence pathway rather than the involvement of specific gene(s) determine the outcome of the soybean resistance to *P. pachyrhizi* (Choi et al. 2008; Goellner et al. 2010; Soria-Guerra et al. 2010a, b; van de Mortel et al. 2007). It remains unknown whether different resistant genotypes employ similar defence mechanisms (Goellner et al. 2010). The identification of multiple transcription factor family members suggests that a complex positive and negative regulation pattern is involved in the host defence against rust infection (Choi et al. 2008).

Ureases were not identified in these large-scale transcript profile studies. It is likely that *GmEu*4 may have a less pronounced involvement in plant responses to fungal infection when compared to other genes. The host phenological stage during infection or the use of different genotypes may have contributed to our findings. In agreement with our results, urease upregulation was observed in *A. thaliana* leaves after 2 h of salicylic acid treatment (available on http://www.ncbi.nlm.nih.gov/geo) (Thibaud-Nissen et al. 2006). Furthermore, in infected soybean plants, a modification in the expression levels of two proteins related to urea metabolism was observed: the Ni-binding urease accessory protein UreG and glutamine synthase (Panthee et al. 2009; Soria-Guerra et al. 2010a). Soria-Guerra et al. (2010a) identified a high number of genes with metabolic-related functions that were upregulated in the resistant *G. tomentella* genotype after infection. The activities of many of the genes involved in metabolic processes are also affected upon pathogen infection and play essential roles in many plant defence responses (Soria-Guerra et al. 2010a). The toxic activity against necrotrophic fungi has been shown to be independent of ureolytic activity (Becker-Ritt et al. 2007). However, it is not clear yet whether the effect of soybean urease on *P. pachyrhizi* is dependent on ureolytic activity.

Although there is insufficient biochemical and molecular information currently available to predict the precise role of *GmEu*4 during fungal infection, the contribution(s) of urease to plant susceptibility to a broad spectrum of fungal pathogens is demonstrated in this study. The transfer of the *GmEu*4 co-suppression characteristic into resistant genotypes and further bioassays may provide new insights into the role of the ubiquitous urease in plant-fungi interaction. Because we assume that urease overexpression, at least in its intact form, may be impossible, the identification and overexpression of the urease fungitoxic peptide is an alternative approach that could reduce the frequency of the co-suppressed phenotype and thereby achieve the desirable increase in the soybean plant resistance to fungi.

## Electronic supplementary material

Below is the link to the electronic supplementary material.

Supplementary Fig 1. GFP expression analyses in transgenic embryogenic tissues and plants. The panels show the following: a) non-transformed somatic embryos; b) green fluorescent areas one week after transformation; c) green fluorescent proliferative somatic embryos four weeks after transformation; d) a non-transformed histodifferentiated embryo (arrow); e) a transgenic histodifferentiated embryo; f) the roots of transgenic and non-transgenic (arrow) plants; g) the leaf of a transgenic plant; h) a non-transgenic seed (arrow) and i) a transgenic seed one day after dormancy was broken. The tissues were derived from the bombardment (b, c, e, f, i) or bombardment/*Agrobacterium* (g) transformation system. GFP expression was detected under blue light using a fluorescence stereomicroscope Olympus^®^, equipped with a BP filter set containing a 488 ηm excitation filter and a 505-530 ηm emission filter. Images were captured using the software QCapture Pro™ 6 (QImaging^®^). Supplementary material 1 (TIFF 1191 kb)

## References

[CR1] Barros PR, Stassen H, Freitas MS, Carlini CR, Nascimento MAC, Follmer C (2009). Membrane-disruptive properties of the bioinsecticide Jaburetox-2Ec: implications to the mechanism of the action of insecticidal peptides derived from ureases. Biochim Biophys Acta.

[CR2] Becker-Ritt AB, Martinelli AH, Mitidieri S, Feder V, Wassermann GE, Santi L, Vainstein MH, Oliveira JT, Fiuza LM, Pasquali G, Carlini CR (2007). Antifungal activity of plant and bacterial ureases. Toxicon.

[CR3] Bradford MM (1976). A rapid and sensitive method for the quantitation of microgram quantities of protein utilizing the principle of protein-dye binding. Anal Biochem.

[CR4] Camargo PO (2010) Estudo da herança de caracteres quali-quantitativos e mapeamento genético de alelos resistentes à Ferrugem Asiática da soja presentes nas PI561356 e PI594754. Dissertation, Universidade Estadual de Londrina

[CR5] Carlini CR, Polacco JC (2008). Toxic properties of urease. Crop Sci.

[CR6] Carlini CR, Oliveira AE, Azambuja P, Xavier-Filho J, Wells MA (1997). Biological effects of canatoxin in different insect models: evidence for a proteolytic activation of the toxin by insect cathepsinlike enzymes. J Econ Entomol.

[CR7] Choi JJ, Alkharouf NW, Schneider KT, Matthews BF, Frederick RD (2008). Expression patterns in soybean resistant to *Phakopsora pachyrhizi* reveal the importance of peroxidases and lipoxygenases. Funct Integr Genomics.

[CR8] Dixon NE, Gazzola TC, Blakeley RL, Zermer B (1975). Letter: Jack bean urease (EC 3.5.1.5). A metalloenzyme. A simple biological role for nickel?. J Am Chem Soc.

[CR9] Doyle JJ, Doyle JL (1987). A rapid DNA isolation procedure for small quantities of fresh leaf tissue. Phytochem Bull.

[CR10] Droste A, Pasquali G, Bodanese-Zanettini MH (2002). Transgenic fertile plants of soybean [*Glycine max* (L) Merrill] obtained from bombarded embryogenic tissue. Euphytica.

[CR12] El-Shemy HA, Khalafalla MM, Fujita K, Ishimoto M (2006). Molecular control of gene co-suppression in transgenic soybean via particle bombardment. J Biochem Mol Biol.

[CR13] Finer JJ, Vain P, Jones MW, McMullen MD (1992). Development of the particle inflow gun for DNA delivery to plant cells. Plant Cell Rep.

[CR14] Follmer C (2008). Insights into the role and structure of plant ureases. Phytochemistry.

[CR15] Follmer C, Wassermann GE, Carlini CR (2004). Separation of jack bean (*Canavalia ensiformis*) urease isoforms by immobilized metal affinity chromatography and characterization of insecticidal properties unrelated to ureolytic activity. Plant Sci.

[CR16] Follmer C, Real-Guerra R, Wasserman GE, Olivera-Severo D, Carlini CR (2004). Jackbean, soybean and *Bacillus pasteurii* ureases: biological effects unrelated to ureolytic activity. Eur J Biochem.

[CR17] Francis KE, Spiker S (2005). Identification of *Arabidopsis thaliana* transformants without selection reveals a high occurrence of silenced T-DNA integrations. Plant J.

[CR18] Goellner K, Loehrer M, Langenbach C, Conrath U, Koch E, Schaffrath U (2010). *Phakopsora pachyrhizi*, the causal agent of Asian soybean rust. Mol Plant Pathol.

[CR19] Goldraij A, Beamer LJ, Polacco JC (2003). Interallelic complementation at the ubiquitous urease coding locus of soybean. Plant Physiol.

[CR20] Graham MY, Weidner J, Wheeler K, Pelow MJ, Graham TL (2003). Induced expression of pathogenesis-related protein genes in soybean by wounding and the *Phytophthora sojae* cell wall glucan elicitor. Physiol Mol Plant Pathol.

[CR21] Homrich MS, Passaglia LMP, Pereira JF, Bertagnolli PF, Pasquali G, Zaidi MA, Altosaar I, Bodanese-Zanettini MH (2008). Resistance to *Anticarsia gemmatalis* Hübner (Lepidoptera, Noctuidae) in transgenic soybean (*Glycine max* (L.) Merrill, Fabales, Fabaceae) cultivar IAS5 expressing a modified Cry1Ac endotoxin. Gen Mol Biol.

[CR59] James VA, Avart C, Worland B, Snape JW, Vain P (2002) The relationship between homozygous and hemizygous transgene expression levels over generations in populations of transgenic rice plants. Theor Appl Genet 104:553–561 10.1007/s001220100745 12582658

[CR22] Jian B, Liu B, Bi Y, Hou W, Wu C, Han T (2008). Validation of internal control for gene expression study in soybean by quantitative real-time PCR. BMC Mol Biol.

[CR23] Karimi M, Inze D, Depicker A (2002). GATEWAY vectors for *Agrobacterium*-mediated plant transformation. Trends Plant Sci.

[CR24] Kohli A, Twyman RM, Abranches R, Wegel E, Stoger E, Christou P (2003). Transgene integration, organization and interaction in plants. Plant Mol Biol.

[CR25] Krajewska B (2009). Ureases I. Functional, catalytic and kinetic properties: a review. J Mol Catal B: Enzymatic.

[CR26] Lechtenberg B, Schuberty D, Forsbachz A, Gils M, Schmidt R (2003). Neither inverted repeat T-DNA configurations nor arrangements of tandemly repeated transgenes are sufficient to trigger transgene silencing. Plant J.

[CR27] Libault M, Thibivilliers S, Bilgin DD, Radwan O, Benitez M, Clough SJ, Stacey G (2008). Identification of four soybean reference genes for gene expression normalization. Plant Genome.

[CR28] Livak KJ, Schmittgen TD (2001). Analysis of relative gene expression data using real-time quantitative PCR and the 2^(-Delta Delta C(T))^ method. Methods.

[CR29] Menegassi A, Wassermann GE, Olivera-Severo D, Becker-Ritt AB, Martinelli AH, Feder V, Carlini CR (2008). Urease from cotton (*Gossypium hirsutum*) seeds: isolation, physicochemical characterization, and antifungal properties of the protein. J Agric Food Chem.

[CR31] Meyer-Bothling LE, Polacco JC (1987). Mutational analysis of the embryo-specific urease locus of soybean. Mol Gen Genet.

[CR32] Miles MR, Levy C, Morel W, Mueller T, Steinlage T, van Rij N, Frederick RD, Hartman GL (2007). International fungicide efficacy trials for the management of soybean rust. Plant Dis.

[CR33] Mulinari F, Staniscuaski F, Bertholdo-Vargas LR, Postal M, Oliveira-Neto OB, Rigden DJ, Grossi-de-Sa MF, Carlini CR (2007). Jaburetox-2Ec: an insecticidal peptide derived from an isoform of urease from the plant *Canavalia ensiformis*. Peptides.

[CR34] Napoli C, Lemieux C, Jorgensen R (1990). Introduction of a chimeric chalcone synthase gene into petunia results in reversible co-suppression of homologous genes in *trans*. Plant Cell.

[CR35] Panthee DR, Yuan JS, Wright DL, Marois JJ, Mailhot D, Stewart CN (2007). Gene expression analysis in soybean in response to the causal agent of Asian soybean rust (*Phakopsora pachyrhizi* Sydow) in an early growth stage. Funct Integr Genomics.

[CR36] Panthee DR, Marois JJ, Wright DL, Narvaez D, Yuan JS, Stewart CN (2009). Differential expression of genes in soybean in response to the causal agent of Asian soybean rust (*Phakopsora pachyrhizi* Sydow) is soybean growth stage-specific. Theor Appl Genet.

[CR37] Polacco JC, Havir EA (1979). Comparisons of soybean urease isolated from seed and tissue culture. J Biol Chem.

[CR38] Polacco JC, Holland MA (1993). Roles of urease in plant cells. Int Rev Cytol.

[CR39] Polacco JC, Winkler RG (1984). Soybean leaf urease: a seed enzyme?. Plant Physiol.

[CR40] Polacco JC, Krueger RW, Winkler RG (1985). Structure and possible ureide degrading function of the ubiquitous urease of soybean. Plant Physiol.

[CR41] Schmidt M, Parrott W (2001). Quantitative detection of transgenes in soybean [*Glycine max* (L.) Merrill] and peanut (*Arachis hypogaea* L.) by real-time polymerase chain reaction. Plant Cell Rep.

[CR42] Schmidt MA, Lafayette PR, Artelt BA, Parrott WA (2008). A comparison of strategies for transformation with multiple genes via microprojectile-mediate bombardment. In Vitro Cell Dev Biol.

[CR43] Shou H, Frame BR, Whitham SA, Wang K (2004). Assessment of transgenic maize events produced by particle bombardment or *Agrobacterium*-mediated transformation. Mol Breed.

[CR44] Sinclair JB, Hartman GL, Hartman GL, Sinclair JB, Rupe JC (1999). Soybean diseases. Compendium of soybean diseases.

[CR45] Soria-Guerra RE, Rosales-Mendoza S, Chang S, Haudenshield JS, Padmanaban A, Rodriguez-Zas S, Hartman GL, Ghabrial SA, Korban SS (2010). Transcriptome analysis of resistant and susceptible genotypes of *Glycine tomentella* during *Phakopsora pachyrhizi* infection reveals novel rust resistance genes. Theor Appl Genet.

[CR46] Soria-Guerra RE, Rosales-Mendoza S, Chang S, Haudenshield JS, Zheng D, Rao SS, Hartman GL, Ghabrial SA, Korban SS (2010). Identifying differentially expressed genes in leaves of *Glycine tomentella* in the presence of the fungal pathogen *Phakopsora pachyrhizi*. Planta.

[CR47] Stebbins NE, Polacco JC (1995). Urease is not essential for ureide degradation in soybean. Plant Physiol.

[CR48] Tang W, Newton RJ, Weidner DA (2007). Genetic transformation and gene silencing mediated by multiple copies of a transgene in eastern white pine. J Exp Bot.

[CR49] Thibaud-Nissen F, Wu H, Richmond T, Redman JC, Johnson C, Green R, Arias J, Town CD (2006). Development of *Arabidopsis* whole-genome microarrays and their application to the discovery of binding sites for the TGA2 transcription factor in salicylic acid-treated plants. Plant J.

[CR50] Torisky RS, Griffin JD, Yenofsky RL, Polacco JC (1994). A single gene (*Eu*4) encodes the tissue-ubiquitous urease of soybean. Mol Gen Genet.

[CR51] Twizeyimana M, Bandyopadhyay R, Ojiambo P, Paul C, Hartman GL (2006) A detached leaf method to evaluate soybean for resistance to rust National Soybean. Rust symposium. In: Proceedings of 2006 National Soybean rust symposium, Saint Louis

[CR52] van de Mortel M, Recknor JC, Graham MA, Nettleton D, Dittman JD, Nelson RT, Godoy CV, Abdelnoor RV, Almeida AM, Baum TJ, Whitham SA (2007). Distinct biphasic mRNA changes in response to Asian soybean rust infection. Mol Plant Microbe Interact.

[CR53] Weatherburn MW (1967). Phenol-hypochlorite reaction for determination of ammonia. Anal Chem.

[CR54] Wiebke-Strohm B, Droste A, Pasquali G, Osorio MB, Bücker-Neto L, Passaglia LMP, Bencke M, Homrich MS, Margis-Pinheiro M, Bodanese-Zanettini MH (2011). Transgenic fertile soybean plants derived from somatic embryos transformed via the combined DNA-free particle bombardment and *Agrobacterium* system. Euphytica.

[CR55] Witte CP, Tiller SA, Taylor MA, Davies HV (2002). Leaf urea metabolism in potato. Urease activity profile and patterns of recovery and distribution of (15)N after foliar urea application in wild-type and urease-antisense transgenics. Plant Physiol.

[CR56] Yin Z, Plader W, Malepszy S (2004). Transgene inheritance in plants. J Appl Genet.

[CR57] Yuan JS, Burris J, Stewart NR, Mentewab AC, Stewart N (2007). Statistical tools for transgene copy number estimation based on real-time PCR. BMC Bioinform.

