# Supplementary figures and images for: Ubiquitous urease affects soybean susceptibility to fungi
Source: Plant Mol Biol. 2012 Mar 1;79(1):75–87. doi: 10.1007/s11103-012-9894-1 (PMC3332383; doi:10.1007/s11103-012-9894-1)

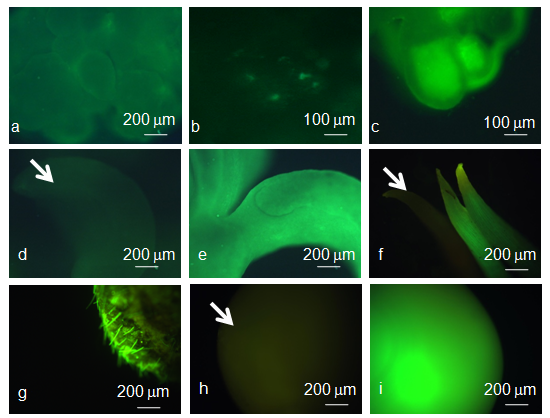

Supplement: Supplementary file 1 — Supplementary Fig 1. GFP expression analyses in transgenic embryogenic tissues and plants. The panels show the following: a) non-transformed somatic embryos; b) green fluorescent areas one week after transformation; c) green fluorescent proliferative somatic embryos four weeks after transformation; d) a non-transformed histodifferentiated embryo (arrow); e) a transgenic histodifferentiated embryo; f) the roots of transgenic and non-transgenic (arrow) plants; g) the leaf of a transgenic plant; h) a non-transgenic seed (arrow) and i) a transgenic seed one day after dormancy was broken. The tissues were derived from the bombardment (b, c, e, f, i) or bombardment/Agrobacterium (g) transformation system. GFP expression was detected under blue light using a fluorescence stereomicroscope Olympus®, equipped with a BP filter set containing a 488 ηm excitation filter and a 505-530 ηm emission filter. Images were captured using the software QCapture Pro™ 6 (QImaging®). Supplementary material 1 (TIFF 1191 kb) [file 11103_2012_9894_MOESM1_ESM.tif]
